# Experimentally constrained network model of hippocampal fast-firing parvalbumin-positive interneurons

**DOI:** 10.1186/1471-2202-13-S1-O5

**Published:** 2012-07-16

**Authors:** Katie A Ferguson, Carey YL Huh, Bénédicte Amilhon, Rosanah Murugesu, Sylvain Williams, Frances K Skinner

**Affiliations:** 1 Physiology, University of Toronto, Toronto, Ontario, M5S 1A1, Canada; 2 Toronto Western Research Institute, University Health Network, Toronto, Ontario, M5T 2S8, Canada; 3 Medicine (Neurology), Physiology, Institute of Biomaterials and Biomedical Engineering, University of Toronto, Toronto, Ontario, M5S 1A1, Canada; 4 Psychiatry, Douglas Mental Health University Institute, McGill University, Montreal, Quebec, H4G 1X6, Canada; 5 Division of Engineering Science, University of Toronto, Toronto, Ontario, M5S 1A1, Canada

## 

The fast-firing properties of parvalbumin-positive (PV+) interneurons, and their extensive connections with neighbouring excitatory neurons, provide them with enormous potential to influence network rhythms and hence behaviour. Thus, it is not entirely surprising that these cells have been implicated in playing a role in a variety of pathologies (e.g. epilepsy [1]). Mathematical modeling allows one to explore the contribution of this population in a simplified setting, and make predictions to guide further experimental work. However, direct links between existing fast-spiking interneuron models and empirically determined cellular intrinsic and network characteristics are unclear, and therefore model predictions can be difficult to interpret in a biological setting. Therefore, we have created a network model of PV+ interneurons that is tied to experimental work on multiple levels, and we use this model to investigate the potential of this population to realize coherent oscillations.

Our PV+ interneurons are represented with an Izhikevich-type model [2], and involve parameter values that are designed to approximate the cell’s intrinsic properties. To determine these parameters, spike characteristics and passive properties were extracted from whole-cell patch clamp recordings of PV+ interneurons in the CA1 region of an intact hippocampal preparation in vitro. Our network model is composed of these individual PV+ cell models, and the network size, architecture, and synaptic properties are chosen to be consistent with those found in the literature. Recordings during emergent network oscillations [3] provided us with information about realistic firing rates and synaptic activity of PV+ interneurons. These firing rates, used in combination with the cell’s intrinsic frequency-current profile, provided physiological constraints on the amount of synaptic current the PV+ cells receive during these spontaneous network oscillations. Under voltage clamp, excitatory post-synaptic current peaks are used in our model as an upper bound on the range of synaptic input. We used this network model to determine whether coherent rhythms could be produced within experimental constraints.

Our model produced intrinsic properties and spiking behaviors which approximated the experimentally determined membrane capacitance, resting membrane potential, threshold potential, spike width, spike peak potential, peak after-hyperpolarizing potential, and amount of adaptation. Model parameters were determined such that the slope of the model’s frequency-current profile and the model rheobase current were within the range of our experimental data. As such, we have produced a network model of PV+ interneurons that has direct links to cellular characteristics with model parameters that have clear biological interpretations. In addition, network simulations of our PV+ interneuron model produced coherent gamma output. Since the firing properties and network architecture of PV+ interneurons puts them in an ideal position to influence network activity, this cell type will likely remain a focus of experimentalists and modelers alike. A model such as ours, with clear links to biology, may be used as a platform to investigate the role of these fast-firing PV+ interneurons in network oscillations and behaviour.
